# Unveiling ADHD’s impact on higher education students: statistics anxiety, attitudes, and statistical literacy

**DOI:** 10.3389/fpsyt.2025.1585601

**Published:** 2025-08-07

**Authors:** Chen Hanna Ryder, Carmit Gal, Miriam Sarid, Anat Klemer

**Affiliations:** ^1^ The Brain & Behavior Research Institute, Western Galilee Academic College, Acre, Israel; ^2^ Department of Education & the Department of Learning Disabilities & Education, Western Galilee College, Acre, Israel; ^3^ Department of Education, Literacy & Innovation in Education Program, Western Galilee College, Acre, Israel

**Keywords:** ADHD, statistical literacy, anxiety, higher education, academic performance, undiagnosed ADHD

## Abstract

**Objective:**

This study investigated the relationship between ADHD and statistical literacy in higher education. We examined how ADHD diagnostic status (diagnosed, suspected undiagnosed, and non-ADHD) relates to statistics anxiety, attitudes, and literacy outcomes, thereby illuminating challenges associated with formal diagnosis and support access. Furthermore, we explored the roles of attitudes, anxiety, ADHD status, and academic degree as predictors of statistical literacy.

**Method:**

We compared three groups among 405 higher education students: students with formally diagnosed ADHD (n = 80), those with suspected undiagnosed ADHD (n = 74), and students without ADHD (n = 251).

**Results:**

Both groups with ADHD reported significantly higher levels of statistics anxiety and less favorable attitudes toward statistics compared to the non-ADHD group. Surprisingly, no significant differences emerged in statistical literacy performance across the three groups. Multiple regression analysis revealed that attitudes toward statistics and academic degree were significant predictors of statistical literacy.

**Conclusion:**

Although students with ADHD experience heightened anxiety and maintain less favorable attitudes toward statistics, they remarkably achieve performance levels comparable to their non-ADHD peers. These findings challenge conventional assumptions about ADHD’s impact on quantitative academic capabilities and highlight the resilience of students with ADHD. The results emphasize the need for targeted interventions focusing on reducing anxiety and improving attitudes while recognizing students’ academic potential.

## 1 Introduction

Statistical literacy represents a fundamental skill in higher education, defined as the ability to understand, critically evaluate, and make informed decisions based on statistical data (1, 2). This competency extends beyond basic numerical understanding, encompassing the capacity to interpret complex quantitative information and engage meaningfully with research findings. As the volume and complexity of data in academic and professional contexts continue to grow, the development of strong statistical literacy has become increasingly crucial for student success across disciplines (3, 4). This multifaceted skill encompasses not only the ability to understand statistical concepts but also the capacity to critically evaluate statistical information presented in various formats, such as graphs, tables, and research reports (2, 4). Students equipped with strong statistical skills are better prepared to engage with research findings, evaluate public health data, and make informed decisions based on quantitative information (4, 5). This capability becomes particularly crucial in contemporary academic contexts, where the ability to discern credible data from misleading statistics has become essential for academic and professional success (2, 4).

Research indicates that many students in higher education struggle with developing statistical literacy, which can significantly impact their academic success and ability to engage with realworld data (4, 6). These challenges become particularly pronounced for students with learning difficulties such as Attention-Deficit/Hyperactivity Disorder (ADHD), who often experience elevated levels of anxiety and negative attitudes towards statistics, potentially affecting their academic achievement (7–10). Recent research by Levy & Stukalin (11) investigated the relationship between ADHD and academic performance in a post-secondary statistics course. Their study revealed a significant indirect link where ADHD was associated with lower self-efficacy, which in turn led to a negative attitude towards statistics and ultimately lower grades. Within the framework of social determinants of health, these academic challenges represent critical factors that may contribute to educational disparities and subsequent health inequities for students with neurodevelopmental conditions such as ADHD.

ADHD represents a significant neurodevelopmental condition characterized by persistent patterns of inattention, hyperactivity, and impulsivity that substantially impact functioning and development (12). The core symptoms of ADHD, including difficulties with sustaining attention, organizing tasks, and regulating activity levels, often persist into adulthood, affecting various aspects of academic and personal life (13). In higher education settings, the prevalence of ADHD has become increasingly significant, with estimates ranging from 2% to 8% (14), and some studies suggesting rates as high as 15.9% (15). However, these figures likely underestimate the true prevalence, as many students with ADHD symptoms may not have received formal diagnosis (16). ADHD often continues to be undiagnosed and not treated until adult life (17) probably because of the difference in symptoms of children and adults. For instance, symptoms of impulsiveness and hyperactivity may decrease with age, but attention deficit remains similar (18). The lack of screening and recognition for students with ADHD who enter higher education and remain without support and proper intervention may affect their academic performance as well as their overall well-being (19). The discrepancy of recognition and screening of ADHD highlights an important social determinant of health: access to diagnostic services and subsequent accommodations varies significantly across different socioeconomic contexts, creating potential disparities in educational outcomes. This undiagnosed population faces a double disadvantage: experiencing the cognitive and emotional challenges associated with ADHD while being ineligible for the formal accommodations and support services available to their diagnosed peers. Recent research highlights the potential scale of this undiagnosed population. For instance, in a sample of 342 Pakistani medical students who had not been previously diagnosed with ADHD, nearly 35% endorsed symptoms consistent with the disorder on a validated screening questionnaire (ASRS), with the inattentive presentation being most common (20). As these students did not undergo a comprehensive clinical evaluation, these figures represent a high-risk population rather than confirmed clinical diagnoses, underscoring the importance of distinguishing between screening results and formal diagnostic assessment. Understanding how these students navigate academic challenges and develop compensatory strategies may provide valuable insights into academic resilience factors that may benefit the broader ADHD population.

Students with ADHD in higher education frequently encounter challenges with academic self-efficacy, personal-emotional adjustment, and organizational skills (13, 21), often resulting in lower academic achievement compared to their peers without the disorder (22). Moreover, students with ADHD often experience lower self-efficacy in academic settings (23), particularly in subjects perceived as challenging (21), such as statistics. This reduced self-efficacy can contribute to more negative attitudes towards statistics, which may in turn affect their engagement with the material and overall performance. Recent studies found that college students with elevated ADHD symptoms consistently report lower levels of academic motivation, which can contribute to more negative attitudes towards challenging subjects such as statistics (24–26).

ADHD has been also found to significantly influence anxiety levels through multiple pathways, especially in academic environments that require sustained attention and complex cognitive processing. Individuals with ADHD often experience heightened anxiety due to cognitive challenges associated with the condition, such as attention deficits and impulsivity (27). The relationship between ADHD, anxiety, and cognitive functioning reflects a complex interplay, as studies have shown that executive functioning and daily life functioning, significantly influence the relationship between ADHD and mood symptoms in university students (28). This interplay is a critical component of the risk factors faced by students with ADHD, potentially affecting their mental health and academic resilience.

The quantitative field, with mathematics as a prime example, presents significant challenges for students with ADHD due to its high cognitive demands. Research indicates that ADHD students consistently report elevated levels of test anxiety (29). Subjects such as mathematics and statistics require sustained attention and complex problem-solving skills, and therefore mathematical anxiety further exacerbates these difficulties. The cognitive challenges associated with ADHD, including difficulties in maintaining focus and managing impulsivity, intensify anxiety in mathematical contexts, often leading to poorer performance (30, 31). Understanding these cognitive-emotional interactions is essential for developing targeted interventions that mitigate these risks and foster resilience among students with ADHD.

Recent research has illuminated critical mechanisms underlying this relationship, revealing that excessive mind wandering and rumination serve as key mediators between ADHD symptoms and anxiety in adults with ADHD (32). This finding underscores the importance of addressing not only the primary symptoms of ADHD but also the secondary cognitive processes that may exacerbate anxiety in academic settings, particularly in statistically oriented courses where sustained attention and sequential processing are crucial. These cognitive processes represent potential targets for intervention to enhance resilience factors in this population.

The relationship between attitudes toward statistics and statistical literacy is well-established in educational research, with particular relevance to students with learning challenges. Studies have consistently shown that positive attitudes towards statistics correlate with higher levels of statistical literacy and academic performance. Research indicates that negative attitudes towards statistics can adversely affect students’ performance in statistical examinations. For instance, Razali’s study (33) found that students’ attitudes had a significant negative effect on their performance in statistics examinations, corroborating earlier findings that negative perceptions of statistics hinder academic success in this subject area (33). Recent research (34) that examined 201 M.A students’ self-efficacy towards statistics and their performance in statistics demonstrated that statistics self-efficacy plays a crucial mediating role between attitudes and statistical literacy. This study suggests that students who perceive statistics as useful and relevant develop greater confidence in their statistical abilities, which in turn enhances their literacy in this domain. This self-efficacy represents a potential resilience factor that could help mitigate the academic challenges faced by students with ADHD. Research by Zhang (35) indicates that self-efficacy positively influences learning motivation, particularly in challenging subjects. Students who believe in their abilities are more likely to engage actively in their learning processes, which is essential for those with ADHD who face additional challenges. This sense of self-efficacy can enhance their motivation to overcome obstacles and persist in their studies of complex subjects such as statistics. As a protective factor, self-efficacy may be especially important for students from underrepresented groups or those lacking formal diagnosis and support.

The availability and effectiveness of academic accommodations significantly impact the motivation of students with learning difficulties. Walker and Test (36) found that self-advocacy skills, which enable students to request necessary accommodations, are crucial for academic success. When students feel supported and have access to appropriate resources, their motivation to engage with challenging material increases. Conversely, lack of understanding or access to accommodations can lead to frustration and decreased motivation, as students may struggle to meet academic demands without necessary support. Access to accommodations represents a key social determinant of educational outcomes and may vary significantly across different socioeconomic contexts, creating potential disparities.

The ability to engage with and comprehend statistical concepts has been found to be related also to cognitive challenges faced by students with ADHD such as executive functions. Executive functions encompass crucial processes including working memory, cognitive flexibility, and inhibitory control (23, 37). Statistical literacy requires skills of sustained attention and complex problem-solving abilities (38). These cognitive challenges manifest specifically in statistical literacy through difficulties in concept comprehension, data interpretation, and method application, potentially compromising academic achievement (39, 40). Research consistently shows that students with ADHD often exhibit significant deficits in these areas (41, 42), which can substantially impair their ability to process and apply statistical information effectively. These cognitive challenges constitute important risk factors that may be particularly pronounced in educational contexts.

Understanding the impact of ADHD whether diagnosed or undiagnosed, on academic performance, has become increasingly crucial for providing appropriate support in higher education (20). Our study examined how ADHD status affects statistical literacy by comparing three groups: students with formally diagnosed ADHD, those suspecting they have ADHD but lack formal diagnosis, and students without ADHD. This approach specifically addresses an underrepresented group in the literature—students with suspected but undiagnosed ADHD - who may face unique barriers to educational success without access to formal accommodations or support services. This population is particularly significant as they may represent a substantial proportion of students with ADHD symptoms who navigate academic challenges without formal support systems.

The current study investigated group differences in statistics anxiety, attitudes towards statistics, and statistical literacy performance, while also examining factors predicting statistical literacy performance - including ADHD status, anxiety levels, attitudes, and demographic variables. This structured approach aimed to deepen our understanding of how ADHD status relates to statistical literacy, potentially informing future educational support strategies. By examining both anxiety and attitudes towards statistics, our study provides a nuanced picture of academic outcomes in this underrepresented population.

Four research questions were examined in the study:

1. What are the differences in statistics anxiety between students with diagnosed and undiagnosed ADHD and non-ADHD students.
2. What are the differences in attitudes towards statistics between students with diagnosed and undiagnosed ADHD and non-ADHD students.
3. What are the differences in statistical literacy between students with diagnosed and undiagnosed ADHD and non-ADHD students.
4. What is the predictive value of statistics anxiety and attitudes towards statistics and ADHD status to statistical literacy?

This investigation seeks to enhance our understanding of how ADHD affects statistical literacy, providing insights that could inform educational approaches. The findings could help educators and institutions develop more effective support strategies for teaching statistics to students across the ADHD spectrum in higher education, with particular attention to those who may lack formal diagnosis or accommodation.

## 2 Materials and methods

### 2.1 Participants

The study included 405 higher education students: 80 with formally diagnosed ADHD, 74 who suspected they had ADHD but were not formally diagnosed, and 251 without ADHD. All participants had completed foundational statistics coursework during their higher education studies; recruitment was conducted at academic institutions where such courses (encompassing both theoretical statistics and applied statistical software training) are mandatory curriculum components at both undergraduate and graduate levels. Chi-square tests revealed significant differences between the three groups in gender distribution (χ2 = 5.91, p <.05), with a higher proportion of diagnosed males (26%) than females (17%) across groups (see **Table 1**). There was also a significant difference in academic degree distribution (χ2 = 7.30, p <.05), with a higher proportion of graduate students in the non-ADHD group (76%) compared to both the diagnosed ADHD and suspected ADHD groups (12% each) (see **Table 1**). A significant difference was found among the three groups in self-reported ADHD symptom frequency (F(2, 402) = 31.66, p <.001). *Post-hoc* comparisons using Tukey’s HSD test indicated that both the diagnosed ADHD group (M = 3.01, SD = 0.85) and the suspected ADHD group (M = 3.24, SD = 0.80) reported significantly higher symptom levels compared to the non-ADHD group (M = 2.57, SD = 0.66; p <.001 for both comparisons). No significant difference in symptom frequency was found between the diagnosed and suspected ADHD groups (p >.05) (see **Table 1**). The mean age was similar across groups (non-ADHD: M = 26.5, SD = 4.62; suspected ADHD: M = 26.4, SD = 4.47; diagnosed ADHD: M = 25.8, SD = 3.17; F(2, 402) = 1.01, p >.05) (see **Table 1**).

**Table 1:** Descriptive statistics of participants.

Variable	Category	No ADHD	Undiagnosed ADHD	Diagnosed ADHD	Chi square / F
Gender	Male	67 (61%)	14 (13%)	29 (26%)	5.91*
	Female	184 (62.4%)	60 (20.3%)	51 (17.3%)	
Academic degree pursued	B.A.	199 (59%)	66 (20%)	72 (21%)	7.30*
	M.A	52 (76%)	8 (12%)	8 (12%)	
Age	Mean	26.5	26.4	25.8	F=1.01
	SD	4.62	4.47	3.17	
ADHD symptoms (self-report)	Mean	2.57^a^	3.24^b^	3.01^b^	31.66***
	SD	0.66	0.80	0.85	

SD represents Standard Deviation. Means with different superscripts differ significantly at p < .05 using Tukey's HSD post-hoc test. *p<.05, ***p<.001.

### 2.2 Instruments

The study employed the following validated assessment tools:

#### 2.2.1 Demographic questionnaire

Participants completed a demographic questionnaire capturing information on gender, academic degree, self-reported ADHD diagnosis status, and self-reported suspected ADHD status.

#### 2.2.2 Hebrew version of statistical anxiety rating scale

The H-STARS (43) was employed to comprehensively assess statistics anxiety and attitudes toward statistics. This 35-item instrument uses a 5-point Likert scale (1-5) measuring agreement levels. Steinberger (44) adapted and validated the H-STARS for Hebrew-speaking pre-service teachers, establishing its psychometric properties for education-related populations.

The H-STARS yields distinct measurements for:

Statistics Anxiety, comprising three subscales:

1. Test and Class Anxiety - Nine items assessing anxiety experienced during statistics learning situations, typically within coursework contexts (e.g., “studying for an examination in a statistics course”). This subscale demonstrated robust internal consistency in the current sample (α=0.88), a finding consistent with the reliability range (α=0.80−0.94) reported in Steinberger’s (44) validation study. 2. Interpretation Anxiety - Four items measuring anxiety arising when “interpreting the meaning of a table in an empirical article” and similar scenarios. The subscale exhibited strong reliability in the current sample (α=0.86), falling within the validated range reported by Steinberger (44). 3. Fear of Asking for Help - Three items evaluating anxiety experienced when approaching statistics instructors or peers (e.g., “asking a fellow student for help in understanding an SPSS output”). This subscale showed excellent internal consistency in the current sample (α=0.86), which aligns with the psychometric properties established by Steinberger (44).

**Attitudes Toward Statistics, comprising three subscales:**

**1. Worth of Statistics -** Six items assessing perceptions of the relevance and value of statistics in personal and professional life (e.g., “Statistics is useful”). This subscale showed excellent internal consistency in the current sample (α=0.92).

**2. Computation Self-Concept -** Five items evaluating self-perceived ability and confidence in understanding and performing statistical computations (e.g., “I have a good intuition for statistics”). This subscale demonstrated strong reliability in the current sample (α=0.89).

**3. Fear of Statistics Teachers -** Five items measuring anxiety related to interacting with statistics instructors (e.g., “Statistics teachers are not approachable”). This subscale had good internal consistency in the current sample (α=0.85).

For analytical purposes, composite scores were calculated for both statistics anxiety (mean rating across all anxiety-related items) and attitudes toward statistics (mean rating across all attitude-related items) for each participant. These composite scores range from 1 (indicating low anxiety/negative attitudes) to 5 (indicating high anxiety/positive attitudes).

#### 2.2.3 Statistical literacy task

A bespoke instrument designed by the researchers assessed fundamental aspects of statistical literacy, reflecting core competencies expected following introductory statistics coursework in higher education. This academic task comprised ten multiple-choice questions evaluating applied comprehension of statistical information. Items required participants to interpret data presented in graphical formats and simple tables (i.e., cross-tabulations and percentages) displaying descriptive statistics, understand frequency concepts, and demonstrate comprehension of statistical significance and hypothesis testing principles (e.g., p-value interpretation). While understanding significance levels was assessed, this represented only one component of a broader task requiring integrated statistical literacy skills.

The instrument underwent content validation by three independent researchers with expertise in statistics education to ensure relevance and clarity at the undergraduate level. The task demonstrated acceptable reliability (Cronbach’s α = 0.67). A representative item asked: “When a researcher examines research hypotheses, what is the significance level they would want to obtain in order to prove their hypotheses (for example, about a difference or relationship)?” followed by multiple response options. Statistical literacy scores were calculated as the sum of correct responses, yielding a range from 0 (lowest performance) to 10 (highest performance).

#### 2.2.4 Adult ADHD self-report scale

The Adult ADHD Self-Report Scale Screener (ASRS-v1.1) was used to assess the frequency of recent ADHD symptoms (45). This study employed the brief 6-item version, which is a validated screening tool designed to capture key symptoms of inattention and hyperactivity-impulsivity based on DSM-IV criteria. Items are rated on a 5-point frequency scale ranging from 1 (“Never”) to 5 (“Very Often”). A representative item asks, “How often do you have difficulty keeping your attention when you are doing boring or repetitive work?”. The scale demonstrated good internal consistency in the current sample (α=0.79). For the purpose of group comparisons (ANOVA) and correlational analyses, a continuous total symptom frequency score was calculated by summing the responses for all six items, yielding a score range of 6 to 30. This score was used to measure the overall level of self-reported ADHD symptoms.

### 2.3 Procedure

The study was approved by the Ethics Certification Board of the College. Data collection was administered by a company of online panel, using “Alchemer” platform for online data collection. The questionnaire was filled in anonymously by individuals who reported they are students, during the second semester of the academic year. Respondents signed an informed consent stating they agreed to participate in the study.

### 2.4 Statistical analysis

Statistical analyses were conducted using SPSS version 28.0.1.1. Descriptive statistics were calculated for demographic variables. Chi-square tests of independence were performed to examine the differences in the distribution of categorical demographic characteristics across the three groups.

To address the first three research questions examining group differences in statistics anxiety, attitudes toward statistics, and statistical literacy, one-way analyses of variance (ANOVAs) were conducted. *Post-hoc* comparisons using Tukey’s HSD test were performed when significant main effects were found.

To examine the fourth research question regarding predictors of statistical literacy performance, a multiple linear regression analysis was conducted. Before the regression analysis, assumptions of linearity, normality, and homoscedasticity were checked.

The level of statistical significance was set at p <.05 for all analyses. Statistics anxiety and attitudes toward statistics were computed as a mean score of the rating for each subscale, resulting in scores of 1 (low) to 5 (high). The score of statistical literacy was calculated as a sum of the correct responses (ranging from 0 to 10).

## 3 Results

To examine differences in statistical anxiety, attitudes, and literacy performance between students with formally diagnosed ADHD (n = 80), suspected but undiagnosed ADHD (n = 74), and students without ADHD (n = 251), we conducted a series of one-way ANOVAs.

### 3.1 Research question 1: differences in statistics anxiety levels

To examine differences in statistics anxiety among the three groups, we conducted one-way ANOVAs. Examination of anxiety levels revealed significant group differences (F = 15.32, p <

.001, η² = .071), with a moderate effect size. *Post-hoc* Tukey’s HSD tests indicated that both the diagnosed ADHD group (M = 3.15, SD = 0.74) and suspected ADHD group (M = 3.12, SD = 0.83) reported significantly higher anxiety compared to the non-ADHD group (M = 2.64, SD = 0.85, p <.001 for both comparisons). No significant differences emerged between the two ADHD (i.e. diagnosed and undiagnosed) groups (p = .97). (See **Table 2**).

**Table 2:** Multiple linear regression analysis for variables predicting statistical literacy.

Predictors	B	SE B	β	t	p	95% CI
Academic (M.A.) degree	1.23	0.27	.22	4.53	<.001	[0.69, 1.76]
Attitudes towards statistics	0.94	0.20	.26	4.75	<.001	[0.55, 1.33]
Statistics anxiety	-0.21	0.14	-.08	-1.54	.12	[-0.48, 0.06]
Suspected ADHD	-0.36	0.29	-.06	-1.24	.22	[-0.94, 0.21]
Diagnosed ADHD	-0.52	0.29	-.09	-1.81	.07	[-1.08, 0.05]

R² = .16, F(5, 398) = 14.83, p < .001. CI, confidence interval.

### 3.2 Research question 2: differences in attitudes towards statistics

As shown in **Table 2**, our analysis of attitudes towards statistics yielded significant group differences (F = 8.45, p <.001, η² = .040), with a small to moderate effect size. The non-ADHD group demonstrated significantly more positive attitudes (M = 3.33, SD = 0.64) compared to both the diagnosed ADHD group (M = 3.06, SD = 0.64, p <.001) and suspected ADHD group (M = 3.03, SD = 0.70, p <.001).

### 3.3 Research question 3: differences in statistical literacy

As shown in **Table 2**, Contrary to our expectations, analysis of statistical literacy scores revealed no significant differences between groups (F = 2.14, p >.05, η² = .011), with a small effect size. Performance levels remained comparable across non-ADHD (M = 6.57, SD = 2.31), suspected ADHD (M = 5.81, SD = 2.09), and diagnosed ADHD groups (M = 5.66, SD = 2.36).

**Figure 1:**
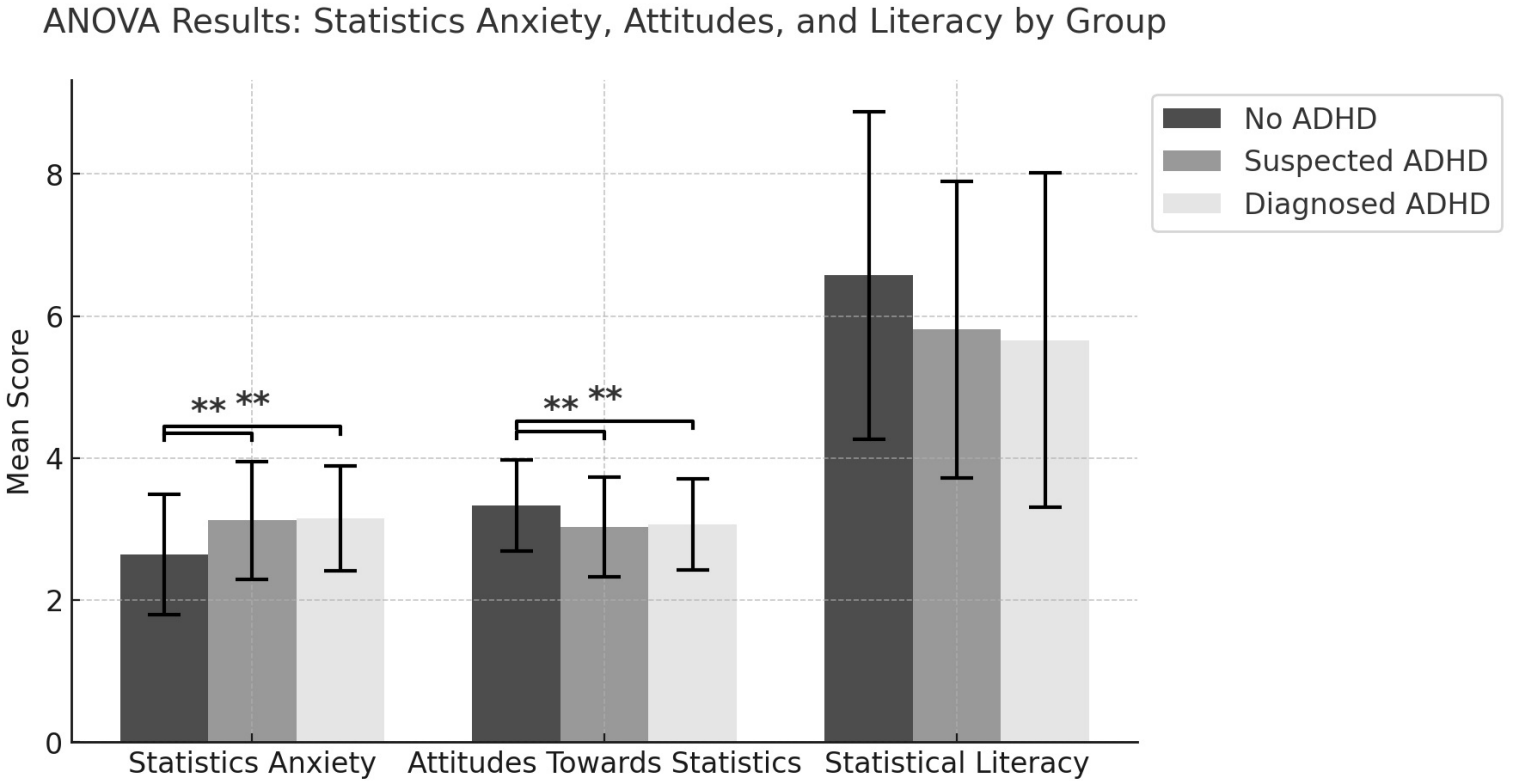
Group differences in statistics anxiety, attitudes towards statistics, and statistical literacy. **p < .01 indicates significant differences between groups.

To summarize the results for the first three hypotheses, both ADHD groups (diagnosed and suspected) reported significantly higher levels of statistics anxiety and more negative attitudes towards statistics compared to the non-ADHD group. However, no significant differences were found in statistical literacy performance between the three groups. **Figure 1** illustrates these patterns of group differences across all three outcome measures.

### 3.4 Research Question 4: Predicting Statistical Literacy Performance

To investigate factors predicting statistical literacy performance, we conducted a multiple linear regression analysis. Preliminary analyses confirmed that assumptions of linearity, normality, and homoscedasticity were met. **Table 2** presents the regression results.

The regression model accounted for 16% of the variance in statistical literacy performance (R² =.16, F(5, 398) = 14.83, p <.001). Academic degree emerged as a significant positive predictor (β = .22, p <.001, 95% CI [0.69, 1.76]), indicating that graduate students demonstrated higher statistical literacy scores compared to undergraduate students. Attitudes towards statistics also significantly predicted performance (β = .26, p <.001, 95% CI [0.55, 1.33]), with more positive attitudes associated with higher statistical literacy scores.

Notably, neither ADHD status (diagnosed: β = -.09, p = .07; suspected: β = -.06, p = .22) nor statistics anxiety (β = -.08, p = .12) significantly predicted statistical literacy performance when controlling for other variables in the model. These findings suggest that while ADHD status is associated with elevated anxiety and more negative attitudes towards statistics, it is not a significant predictor of statistical literacy performance when accounting for academic level and attitudes.

## 4 Discussion

This investigation into the relationships between ADHD, statistical anxiety, attitudes towards statistics, and statistical literacy score in higher education reveals a complex and nuanced picture, offering insights that both challenge and extend our current understanding of these interconnections.

Our findings provide critical insights into how neurodevelopmental conditions impact academic performance and emotional experiences in statistical literacy, while also highlighting unexpected patterns of resilience and capability among students with ADHD. These conditions were examined among self-reported students with ADHD compared to students who have never been diagnosed but reported themselves as suspected having status of ADHD and compared to students without ADHD. This three-group comparison enabled us to examine not only students with ADHD, but also a group neglected in research that may face double risk in higher education: living with ADHD symptoms while never being diagnosed, thus lacking access to academic or emotional support that students with disclosed ADHD are eligible to receive. This focus on the underrepresented group of suspected but undiagnosed ADHD students offers valuable perspective on social determinants of health and educational outcomes with broad global implications. Our results revealed a pronounced pattern of elevated statistics anxiety among both diagnosed and undiagnosed ADHD groups compared to non-ADHD peers. This finding extends beyond the established research on anxiety in ADHD populations by illuminating the specific manifestation of anxiety in statistical literacy contexts. The heightened anxiety levels we observed emerge from multiple combinations of factors, creating a complex web of cognitive and emotional challenges unique to statistical literacy. The relationship between ADHD and statistics anxiety can be understood through recent research by Kandeğer et al. (32), who demonstrated that ADHD symptoms are indirectly associated with anxiety through excessive mind wandering and rumination. In statistical literacy contexts, where sustained attention and sequential processing are essential, these cognitive patterns may become particularly problematic. The cognitive demands inherent in statistical tasks particularly challenge students with ADHD due to their difficulties with attention regulation and executive functioning (46–48). These cognitive impairments, characterized by struggles with working memory, cognitive flexibility, and inhibitory control (41, 42), create cascading effects in statistical literacy. Specifically, students face difficulties maintaining attention during extended statistical calculations, challenges switching between different statistical concepts and procedures, problems inhibiting irrelevant information while focusing on key statistical principles and struggles with organizing, and sequencing multi-step statistical problems. These specific cognitive challenges represent important risk factors that definitively impact students with ADHD in academic environments worldwide.

These ongoing cognitive challenges often lead to decreased self-efficacy and heightened self-doubt, which research has shown can trigger reduced motivation and increased anxiety in academic settings (49, 50). Evidence has revealed (28) that this relationship becomes cyclical, as executive functioning difficulties not only contribute to anxiety but can also be exacerbated by it, creating a complex feedback loop where cognitive challenges increase anxiety, which in turn further impairs cognitive functioning. Notably, our finding that anxiety levels were similarly elevated in both diagnosed and suspected ADHD groups provides empirical support for these complex relationships. The comparable anxiety levels suggest that these cognitive-emotional cascades occur regardless of formal diagnosis, highlighting the importance of supporting students who experience ADHD symptoms even without official diagnosis. This undiagnosed group may represent high-risk population in the academic setting, as despite their ADHD symptoms, they do not receive academic or emotional support when needed, throughout their academic studies. This disparity in access to support services represents a critical social determinant of educational outcomes that clearly exacerbates existing inequities for this underrepresented population.

Our empirical investigation revealed a clear pattern wherein both diagnosed and suspected ADHD groups demonstrated markedly more negative attitudes toward statistics compared to their non ADHD peers. This finding illuminates the complex interplay between cognitive processing, emotional responses, and academic self-perception in statistical education. The striking similarity in attitudinal patterns between diagnosed and suspected ADHD groups suggests that ADHD-related challenges influence statistical attitudes regardless of formal diagnostic status. These attitudinal patterns represent a consistent phenomenon that transcends specific educational contexts.

Research demonstrates that statistical self-efficacy—one’s belief in their ability to succeed in statistical tasks plays a crucial mediating role between attitudes and actual statistical literacy (34). For students with ADHD, this relationship becomes particularly significant, as evidenced by our findings of consistently more negative attitudes in both ADHD groups. Building on previous research (21, 23), regarding reduced academic self-confidence in ADHD populations, our findings demonstrate more negative attitudes toward statistics among both diagnosed and suspected ADHD groups compared to their non-ADHD peers. Such negative attitudes may lead to avoidance of statistical tasks, which in turn reduces opportunities to experience success, creating a self-perpetuating cycle of academic disengagement. While our study did not directly measure avoidance behaviors, the negative attitudes we observed toward statistics suggest potential implications for students with ADHD in statistical learning contexts. These negative attitudes represent a definitive risk factor that compromises academic achievement if not addressed through appropriate interventions.

The cognitive demands of statistical computation present unique challenges for students with ADHD, as demonstrated by our findings of more negative attitudes in both ADHD groups. This pattern aligns with previous research (31), which documents elevated anxiety levels and negative attitudes among college students with ADHD, particularly toward tasks requiring sustained attention and sequential processing—both essential components of statistical literacy tasks. These task-specific challenges highlight the importance of understanding how different academic domains present distinct barriers for students with ADHD.

A particularly intriguing aspect of our findings is the similarity between diagnosed and suspected ADHD groups in their statistical attitudes, with both groups demonstrating equally negative attitudes compared to their non-ADHD peers. This parallel pattern suggests that the mere perception of having ADHD symptoms, rather than formal diagnosis alone, may influence one’s approach to challenging academic subjects. Our observation gains further support from recent research (24), which reveals that students who report ADHD symptoms, whether officially diagnosed or not, consistently show lower academic motivation levels, especially in complex subjects like statistics - a finding that mirrors the attitudinal similarities we found between our diagnosed and suspected ADHD groups. The similarity of both groups (e.g. self-reported ADHD and suspected ADHD) on the ADHD symptoms scale provides further support to these findings, showing that both groups exhibit symptoms of ADHD while studying in higher education. The similarity between those groups may provide additional support to the link between symptoms of ADHD and their statistics anxiety and attitudes towards statistics. This finding conclusively demonstrates the importance of addressing the needs of students with suspected ADHD as a distinct and underrepresented population facing unique barriers to academic success.

The relationship between motivation and academic performance is particularly relevant for students with ADHD. Walker and Test (36) found that self-advocacy skills, which enable students to request necessary accommodations, are crucial for academic success. When students with ADHD feel supported and have access to appropriate resources, their motivation to engage with challenging statistical material increases. Conversely, lack of understanding or access to such support can lead to frustration and decreased motivation, as these students may struggle to meet academic demands without necessary assistance. Access to these educational provisions represents a significant social determinant of educational outcomes that creates a clear barrier between diagnosed and undiagnosed students.

Perhaps most surprisingly, our analysis revealed a striking disconnect between attitudes and performance: despite both ADHD groups showing significantly more negative attitudes toward statistics, we found no significant variation in statistical literacy performance across the three groups. This unexpected finding challenges conventional assumptions about ADHD’s impact on academic capability and highlights the complex relationship between neurodevelopmental conditions and academic achievement. While our results show that students with ADHD perceive statistics more negatively than their non-ADHD peers, their equal performance on statistical literacy tasks suggests these negative self-perceptions may not reflect their actual capabilities. This remarkable pattern of achievement despite significant attitudinal and anxiety-related challenges demonstrates an important resilience factor that demands greater attention in research on ADHD and academic performance.

The absence of significant differences in statistical literacy score across the three groups challenges conventional wisdom regarding ADHD’s academic impact while illuminating complex relationships between cognitive processing and adaptation. This finding may indicate that students with ADHD cultivate sophisticated adaptive strategies to overcome their executive function challenges (51) enabling them to achieve comparable academic outcomes when provided with appropriate support systems (52). Remarkably, despite documented executive function deficits (38), these students appear to leverage intact or potentially enhanced capabilities in other cognitive domains, developing alternative pathways for statistical problem-solving. This adaptive pattern demonstrates significant cognitive flexibility and underscores the importance of recognizing diverse learning approaches in academic achievement. These compensatory strategies represent definitive resilience factors that help students with ADHD overcome the cognitive and emotional challenges they face in academic environments.

The comparable performance levels across groups may point to the need of support systems in higher education for students with ADHD students that can foster their statistical literacy potential (52, 53). These findings emphasize the critical need for developing comprehensive support approaches that incorporate both anxiety management and skill development components. The effectiveness of such support systems represents a universal factor that can be implemented in educational environments globally.

The implementation of therapeutic strategies to reduce anxiety, stress, and statistics anxiety in learning environments has proven essential for student success. Research demonstrates that therapeutic approaches such as the integration of structured mindfulness techniques and targeted anxiety-reduction methods can potentially alleviate academic distress and enhance performance among students with ADHD-related challenges (54, 55). These interventions focus on developing practical coping mechanisms that help students manage both general anxiety and subject-specific anxiety during statistical tasks, enhancing cognitive engagement and academic performance. By addressing both generalized and statistics-specific anxiety through various anxiety reduction techniques, such support strategies could create a more comprehensive framework for helping these students succeed. These therapeutic approaches represent concrete, implementable strategies that can be adopted across educational institutions worldwide.

The development of targeted interventions addressing students’ confidence and attitudes toward statistics emerges as particularly crucial in light of findings regarding the mediating role of statistics self-efficacy (30). While our research demonstrates comparable performance levels in statistical literacy across groups, we found significantly higher levels of statistics anxiety and more negative attitudes toward statistics among both diagnosed and suspected ADHD students, suggesting that support programs should prioritize emotional support, anxiety reduction, and improvement of self-efficacy in learning statistics. Though the comparable performance levels may reflect the effectiveness of existing academic support services that ADHD students already receive, the literature points to opportunities for enhancing these services. Specifically, research suggests incorporating working memory enhancement strategies, organizational tools for complex statistical procedures, and attention management techniques during statistical problem-solving (42), creating a more comprehensive support framework.

Moreover, the striking similarity in patterns of anxiety and attitudes between diagnosed and suspected ADHD groups underscores the importance of extending such comprehensive support to students who self-identify with ADHD symptoms, even without formal diagnosis. While our findings indicate no statistically significant differences between these two groups on the measured outcomes (symptom severity, anxiety, attitudes, and literacy), this very similarity illuminates a critical issue of academic equity. The undiagnosed group achieves comparable statistical literacy scores despite reporting equivalent levels of statistics anxiety and negative attitudes toward statistics as their diagnosed peers, yet they navigate these challenges without access to the formal accommodations, support services, or potential therapeutic assistance typically available following diagnosis [see Introduction]. This suggests they may operate under significantly greater psychological strain, relying heavily on potentially taxing self-developed compensatory strategies and self-regulation. This interpretation aligns with evidence suggesting that undiagnosed individuals may face greater long-term academic challenges compared to those receiving appropriate support (56). Consequently, this group of students represents an important population facing a unique ‘double risk’ within learning environments: experiencing ADHD-related challenges while simultaneously lacking the institutional safety net designed to mitigate these difficulties. Notably, in the context of Israeli higher education, formally diagnosed students are typically eligible for specific accommodations, such as extended time on examinations and supplemental support hours facilitated through the Dean of Students office, resources generally unavailable to their undiagnosed peers. The comparable performance observed in our study on a task reflecting undergraduate-level competency, despite this disadvantage in support access, might point to inherent resilience or particularly effective compensatory skills within this sample. However, the significant emotional toll reflected in their high anxiety and negative attitudes suggests that this performance parity may come at a substantial hidden cost. Recognizing this is crucial, as facilitating access to formal diagnosis and subsequent support holds considerable potential. Interventions targeting not only study skills but specifically their statistics anxiety and attitudes could alleviate their psychological burden, enhance self-efficacy, and potentially enable these undiagnosed students to unlock performance capabilities beyond the compensatory levels observed currently—perhaps even allowing them to surpass the outcomes of diagnosed peers receiving standard accommodations alone. Therefore, encouraging undiagnosed students experiencing these difficulties to seek formal assessment is profoundly important, as the resulting eligibility for accommodations could improve academic performance and potentially foster more positive attitudes toward challenging subjects like statistics. Addressing the unique needs of this underrepresented population is essential for promoting educational equity and reducing disparities in academic outcomes.

Regarding the limitations of the study, several methodological considerations warrant attention when interpreting these findings. First, the cross-sectional design provides valuable insights into associations but inherently limits the ability to establish causal relationships between the variables investigated.

Furthermore, reliance on self-report measures introduces potential reporting biases. This pertains to the assessment of ADHD diagnostic status (self-reported formal diagnosis or suspected status), symptom severity (via the brief ASRS-6 screener), and statistics anxiety and attitudes (via HSTARS). While the ASRS-6 was selected as a standardized symptom frequency measure suitable for all participant groups, particularly avoiding redundant diagnostic procedures for diagnosed students and accommodating potential aversion to formal assessment among the suspected group, it cannot provide a clinical diagnosis or detailed symptom profiles (45). Future research could be strengthened by incorporating objective measures or multi-informant data.

A further methodological consideration relates to the online, unsupervised administration of the study measures. As participants completed the questionnaires at a time and place of their choosing, this introduces a potential confound for the statistical literacy task, as the possibility of participants seeking external assistance cannot be entirely ruled out. However, it is important to note several design features that were implemented to mitigate this risk. First, participants were explicitly instructed to complete the task independently, with the stated goal of assessing their genuine and natural performance. Second, the statistical literacy task was specifically designed to assess applied knowledge of statistical concepts and SPSS outputs, requiring a level of understanding that would be difficult to obtain from non-professional sources. Third, the ‘Alchemer’ online platform used for data collection incorporates technical safeguards that hinder cheating; it prevents the direct copying and pasting of questions or graphical elements to external sources. Moreover, the task utilized interactive graphical models that required direct user manipulation to interpret, a feature designed to increase cognitive engagement and demand professional knowledge acquired in statistics courses. Despite these mitigating measures, we acknowledge that this limitation highlights the value of future research employing more ecologically valid and controlled assessments, such as performance on supervised, in-class examinations or standardized tests. Such high-stakes environments may more accurately reveal the impact of ADHD symptoms on academic performance in quantitative domains.

Regarding instrumentation specific to statistical literacy, the assessment relied on a brief, 10-item custom-built task. Although this task was carefully constructed and content-validated to assess foundational competencies relevant to the expected academic level (as detailed in Methods 2.2), its modest reliability in our sample (α = 0.67) and lack of extensive external psychometric validation should be noted. The task’s brevity and specific item characteristics might not fully capture the complexity of statistical literacy. Consequently, we concur that further research utilizing more psychometrically robust statistical literacy measures is warranted.

Additionally, the study did not measure several potentially influential variables. These include specific accommodation use among diagnosed students, co-occurring conditions, medication status, and therapeutic interventions for ADHD. The protocol deliberately excluded the collection of data on medication or treatment status; this decision aimed to prioritize participant comfort and privacy, particularly for individuals potentially hesitant to disclose sensitive health information, thereby intending to maximize recruitment and honest reporting across groups by minimizing intrusive questions. Nonetheless, the resulting lack of data on medication/treatment status is acknowledged as a significant limitation, given its potential influence on symptom expression or performance. Regarding other life stressors, these were also omitted from measurement; while such factors could undoubtedly be influential, adding extensive stressor questionnaires was deemed impractical due to participant burden considerations within the study’s length and demands, representing a necessary methodological trade-off.

Finally, considerations related to the sample itself are warranted. While recruitment aimed to minimize selection bias (participants were recruited from institutions where statistics is mandatory) and the influence of academic degree level was controlled analytically, caution is needed regarding the specific sample characteristics (e.g., the combination of B.A. and M.A. students) and the generalizability of findings beyond the particular institutional context. While our sample size (N=405) provided reasonable statistical power for the main analyses conducted, larger sample sizes would enhance the robustness of the findings and the ability to detect smaller effects. Moreover, the lack of data collected on other potentially relevant demographic variables (e.g., socioeconomic status, ethnicity) may limit the generalizability or interpretation of our findings regarding the broader population of higher education students.

Looking toward future directions, longitudinal studies are particularly crucial for tracking the development of attitudes and their influence over time. This approach becomes especially important given research demonstrating that while certain aspects of statistical learning remain intact in ADHD individuals, related cognitive functions show impairment, suggesting complex adaptation processes that warrant further investigation. Building on these findings, understanding how positive attitudes toward statistics develop and influence both performance and academic advancement could provide crucial insights for designing effective educational interventions. Furthermore, the cognitive and emotional mechanisms underlying statistical learning in ADHD students deserve particular attention, especially considering the emphasis on statistical literacy’s growing importance in contemporary data-driven environments (4). Further research could also benefit from analytical approaches beyond group comparisons, such as examining the continuous relationship between symptom scores and literacy, or conducting detailed item-level analyses of the statistical literacy task. Exploration into the efficacy of different intervention approaches would also be valuable; for example, comparing interventions combining statistical coping strategies with anxiety reduction techniques versus those focusing solely on emotional or cognitive aspects. Examining how social determinants of health, including access to diagnosis and support, impact outcomes across diverse contexts also remains an important avenue.

Our investigation reveals a compelling paradox in statistical learning among higher education students: while those with ADHD, both formally diagnosed and self-suspected, experience significantly higher levels of statistical anxiety and maintain more negative attitudes toward statistics compared to their non-ADHD peers, they nevertheless achieve comparable statistical literacy performance. This remarkable finding not only demonstrates these students’ substantial cognitive potential and resilience in overcoming emotional and attitudinal challenges but also fundamentally challenges prevailing assumptions about ADHD-related academic limitations, illuminating the sophisticated compensatory strategies these students develop to succeed. This pattern of resilience despite significant challenges offers important insights for understanding how students with ADHD navigate academic environments and develop effective coping mechanisms that can be applied globally.

In-depth analysis of our findings yields critical insights into these dynamics, revealing that attitudes toward statistics and academic degree level, rather than ADHD status or anxiety levels, serve as the primary predictors of statistical literacy performance. This discovery underscores the paramount importance of nurturing positive attitudes and building confidence in statistical abilities. Particularly telling is the marked underrepresentation of ADHD students in advanced degree programs (12%), suggesting that emotional and attitudinal barriers, rather than cognitive limitations, constitute the primary obstacles to academic advancement. This underrepresentation highlights an important educational disparity that reflects broader social determinants affecting access to advanced education for students with ADHD.

These compelling findings demand a fundamental reconceptualization of traditional educational approaches that typically emphasize cognitive support while overlooking the crucial emotional and attitudinal dimensions of learning. Although current educational frameworks enable ADHD students to achieve performance parity with their peers, this success often comes at considerable psychological cost, highlighting the pressing need for a paradigm shift in educational support strategies to ensure sustainable academic success without compromising psychological well-being.

Addressing these psychological costs is essential for promoting not only academic achievement but also overall health and well-being for students with ADHD.

To address these interconnected challenges systematically, we propose an evidence-based, three tiered intervention strategy that addresses both risk and resilience factors for this underrepresented population:

1. Reducing statistical anxiety through comprehensive implementation of evidence-based stress management techniques, incorporating mindfulness practices, targeted relaxation exercises, and specialized interventions for test anxiety. These approaches target key risk factors that may compromise academic performance and psychological well-being.
2. Enhancing self-efficacy through systematic development of positive attitudes toward statistics, positioning it as an indispensable tool for academic and professional advancement, while methodically strengthening students’ confidence in their statistical literacy. These interventions aim to foster resilience factors that can help students overcome the challenges they face.
3. Broadening support accessibility through carefully designed interventions that encompass both diagnosed and self-identified ADHD populations, ensuring support allocation based on symptomatic needs rather than diagnostic status alone. This approach addresses a critical social determinant—access to support services—that creates disparities between diagnosed and undiagnosed students with ADHD symptoms.

These findings collectively underscore the imperative for creating more inclusive, empathetic, and psychologically informed educational environments. By comprehensively addressing the cognitive, emotional, and attitudinal dimensions of learning among students with ADHD, educational institutions can foster both academic excellence and positive learning experiences. Such transformative approaches prove essential for building genuine educational equity and resilience in higher education, ultimately enabling all students, regardless of their neurodevelopmental profiles, to fully actualize their academic potential and thrive in their educational journey. This focus on inclusive educational practices represents an important approach to addressing social determinants of health and promoting equity for underrepresented populations in higher education globally.

In conclusion, our study provides clear evidence that students with ADHD—both diagnosed and undiagnosed—demonstrate significant resilience, achieving comparable statistical literacy performance despite experiencing heightened anxiety and maintaining more negative attitudes toward statistics. The striking similarities in these challenges between diagnosed and suspected ADHD groups highlight the importance of recognizing and addressing the needs of the substantial population of students who navigate ADHD symptoms without formal diagnosis. Furthermore, our findings definitively identify attitudes toward statistics and academic degree level, rather than ADHD status or anxiety per se, as the primary predictors of statistical literacy performance, emphasizing the critical importance of targeting emotional and attitudinal dimensions in educational support. These findings underscore the need for enhanced institutional awareness of statistics anxiety and negative attitudes, alongside the implementation of targeted interventions, such as the three-tiered strategy proposed. Such a combined approach, directly targeting the emotional and attitudinal factors identified as critical predictors while facilitating access to support through established pathways including formal assessment, holds significant promise for improving the learning experience, mitigating anxiety, and ultimately elevating academic performance for all students navigating ADHD symptoms.

## Data Availability

The datasets presented in this article are not readily available due to data privacy promised to the participants. Requests to access the datasets should be directed to miris@wgalil.ac.il.
